# Multiparametric Quantitative Analysis of Photodamage to Skin Using Optical Coherence Tomography

**DOI:** 10.3390/s23073589

**Published:** 2023-03-29

**Authors:** Han Tang, Chen Xu, Yakun Ge, Mingen Xu, Ling Wang

**Affiliations:** 1 School of Automation, Hangzhou Dianzi University, Hangzhou 310018, China; 2 Key Laboratory of Medical Information and 3D Bioprinting of Zhejiang Province, Hangzhou 310000, China

**Keywords:** ultraviolet, artificial skin, optical coherence tomography, roughness, scattering coefficient, texture feature

## Abstract

Ultraviolet (UV) irradiation causes 90% of photodamage to skin and long-term exposure to UV irradiation is the largest threat to skin health. To study the mechanism of UV-induced photodamage and the repair of sunburnt skin, the key problem to solve is how to non-destructively and continuously evaluate UV-induced photodamage to skin. In this study, a method to quantitatively analyze the structural and tissue optical parameters of artificial skin (AS) using optical coherence tomography (OCT) was proposed as a way to non-destructively and continuously evaluate the effect of photodamage. AS surface roughness was achieved based on the characteristic peaks of the intensity signal of the OCT images, and this was the basis for quantifying AS cuticle thickness using Dijkstra’s algorithm. Local texture features within the AS were obtained through the gray-level co-occurrence matrix method. A modified depth-resolved algorithm was used to quantify the 3D scattering coefficient distribution within AS based on a single-scattering model. A multiparameter assessment of AS photodamage was carried out, and the results were compared with the MTT experiment results and H&E staining. The results of the UV photodamage experiments showed that the cuticle of the photodamaged model was thicker (56.5%) and had greater surface roughness (14.4%) compared with the normal cultured AS. The angular second moment was greater and the correlation was smaller, which was in agreement with the results of the H&E staining microscopy. The angular second moment and correlation showed a good linear relationship with the UV irradiation dose, illustrating the potential of OCT in measuring internal structural damage. The tissue scattering coefficient of AS correlated well with the MTT results, which can be used to quantify the damage to the bioactivity. The experimental results also demonstrate the anti-photodamage efficacy of the vitamin C factor. Quantitative analysis of structural and tissue optical parameters of AS by OCT enables the non-destructive and continuous detection of AS photodamage in multiple dimensions.

## 1. Introduction

The skin is the largest organ in the human body and is an important protective barrier against physical, chemical, and microbial attacks [1]. As the outermost tissue of the body, its long-term exposure to UV irradiation induces a series of skin photodamages [2]. Two UV bands contribute to the photodamage of the skin, namely, UVA (320 nm~400 nm) and UVB (280 nm~320 nm) [3]. Among the two bands, UVA outperforms UVB in penetrability and is believed to be the main cause of severe skin photodamage and aging. Long-term exposure to UVA irradiation can cause skin aging and even skin diseases such as cancer [3,4]. The detection of photodamage and the establishment of photodamage models are important for the study of the pathological mechanisms of the skin caused by UV irradiation and for the treatment and prevention of photodamage. Currently, animal models, human models, and in vitro models serve as the main sources for skin photodamage research. Among them, animal models [3,5] have intact skin structures and can simulate some photodamage models, but an interspecific difference exists between animal models and human skin. Human models, on the other hand, are generally only used to simulate acute skin photodamage, as ethical issues limit the ability to expose normal humans to UV irradiation for long periods of time [6]. Therefore, a combination of ethical and biocompatibility constraints has led to the growing popularity of in vitro cultured AS models [6,7,8,9,10], which are widely used in biotechnology and biomedical research. With a high degree of similarity to human tissue in terms of morphological structure, lipid structure, and biochemical reactions, in addition to the absence of ethical issues, these AS models are gradually replacing animal models as the main tool for the drug and cosmetic industry development and in vitro testing, and are being used in dermatopathology, pharmacology, and toxicology studies [11,12,13]. The morphological structure, optical properties, and cellular state of AS change during UV irradiation as photodamage accumulates, and these factors reveal the degree of photodamage and have the potential to guide research in UV protection and sunburn repair. Currently, invasive experiments act as the gold standard for the evaluation of skin photodamage [14,15,16]. As far as we know, there are no elements to support a non-invasive, continuous monitoring method to facilitate the assessment of mechanisms underlying skin photodamage.

OCT is a non-destructive, three-dimensional imaging technique with advantages such as cross-sectional, high-resolution, and real-time tissue imaging [17,18,19,20], and it is widely used in skin detection studies [6,21,22,23,24]. In studies about skin diseases or damage, OCT is used in animal experiments. Kulikov et al. showed that OCT images of photoaging in animal models correlated well with histological findings, validating the feasibility of the continuous OCT detection of photodamage [25]. However, only acute UV damage models were established that lacked the continuous observation of samples as well as highly specific immunohistochemical and immunofluorescence techniques. Wu et al. analyzed the optical properties and morphological changes in UVB-induced skin cancer processes and demonstrated that OCT is a useful tool for monitoring UVB-induced skin cancer processes [26]. However, the skin samples of mice were severely deformed during the carcinogenesis process. Whether this method is applicable to most skin disease detection processes still needs further validation. Liu et al. showed that OCT can be used to detect and assess different doses of narrowband UVB and broadband UVB-induced photodamage in mouse skin tissue [3]. There are species differences between animals and humans, and further investigation is needed to determine whether OCT is applicable to human skin. OCT techniques have also been adopted in skin research on human skin. Sieglinde et al. applied OCT with confocal laser scanning microscopy to characterize structural and age-related changes in human skin and verified that OCT images can reflect structural differences between older and younger skin [27]. However, studies on the skin aging process are lacking, and it is still unknown whether OCT can identify subtle changes in the skin aging process. Furthermore, the methods proposed in these studies mainly reflect photodamage through changes in the morphological structure (thickness) of skin samples. Wu et al. used OCT to continuously detect the epidermal thickness and attenuation coefficient of photoaging and aged skin, demonstrating that OCT can be used to improve and monitor aging skin treatments [28]. Boone et al. validated that HD-OCT can be used to qualitatively and quantitatively assess the skin from the skin surface to the superficial reticular dermis, allowing for the observation of age-related differences in different skin layers and offering a new possibility to test the efficacy of different cosmetic products [29]. Studies on skin pharmacology, pathology, and toxicology also need to focus on the cellular state within the skin. The focus of cosmetic and sunscreen skin care products is on the smoothness of the skin surface. There is therefore an urgent need for a multidimensional approach to the analysis of photodamage.

Many studies have reported OCT signal process techniques to evaluate or quantify the parameters of a sample, as OCT data reveal more information about the sample than merely its 3D structure [13,30,31,32,33]. Schmitt et al. obtained internals of the upper surface of the skin based on B-scan images and characterized the morphological features of the skin tissue [13]. However, direct quantification of skin roughness cannot be achieved because it introduces accuracy errors and strong randomness. The two-dimensional images can only characterize part of the skin. Askaruly et al. performed boundary identification on skin phantom OCT images and calculated the skin roughness according to the definition of part 2 of the ISO 25178 standard [30]. The reliability of the roughness measurements was demonstrated by comparing the results with those from the PRIMOS skin measurement device. Zhao et al. utilized OCT A-scan signal analysis and an adaptive algorithm to realize the quantification of the roughness of AS samples for a quality check [31]. In addition, tissue optical characterization is widely used in OCT biological tissue image pathology. Liu et al. obtained the relationship between the scattering attenuation coefficient and skin damage by measuring the degree of damage from different UV rays on the back skin of mice through the classical exponential curve fitting method [32]. However, their attenuation coefficient calculation method requires a large number of valid pixel points to be fitted, and the thin thickness of artificial skin makes it difficult to obtain accurate results by exponential fitting. Choi et al. used a single-scattering model depth-resolution algorithm to calculate attenuation coefficients from the depth of the anatomical plane via OCT [33], by which the attenuation coefficients at each pixel in the depth direction can explain the variation in optical properties in heterogeneous multilayered tissues and distinguish phagocytic plaque types by their variations. On account of the heterogeneous distribution of cells within the artificial skin and the inherent errors in the depth-resolution algorithm, there are still problems to overcome in order to accurately quantify the attenuation coefficient of AS. Tissue classification and segmentation can also be performed based on OCT images, and different algorithms, including artificial intelligence, techniques have been used [34,35,36,37]. Among these studies, Adabi et al. analyzed the optical properties (i.e., attenuation coefficient) and texture features of OCT-segmented images. The parameters of healthy samples were compared with those of cancerous skin samples and classified using a linear support vector machine (SVM) whereby a high accuracy was obtained [35]. Neural networks have been used in the OCT image segmentation of mouse skin, showing its advantages in terms of speed and accuracy [37]. Based on the above studies, OCT data analysis has the potential to provide a long-term, multidimensional evaluation of AS in UV irradiation experiments in both morphology and bio-activity aspects.

In this study, UVA was used to irradiate artificial skin to establish the photodamage model. The AS was continuously and non-destructively examined by OCT. The structural parameters and tissue optical parameters of AS were quantified based on OCT interferometric signals. The feasibility of assessing photodamage via the multiparametric quantitative OCT analysis of AS was verified by comparing the results with those from tissue section microscopy and bioactivity assay experiments. We quantified and analyzed the change patterns of parameters of the conventional cultured AS and the benign control model with a vitamin C (VC) anti-photodamage factor added to the culture medium and the photodamage model under the same UV irradiation dose. The photodamage caused by UV irradiation to the AS was also assessed in a multidimensional manner based on the intergroup differences between the three groups. The aim of this study was to investigate the changes in skin parameters due to photodamage in AS using OCT. This provides a new method for the non-destructive detection of photodamage in AS, and it will pave the way to further understand the mechanism of skin photodamage and for future research on anti-photodamage products.

## 2. Materials and Methods

### 2.1. Modeling AS Photodamage

**Figure 1:**
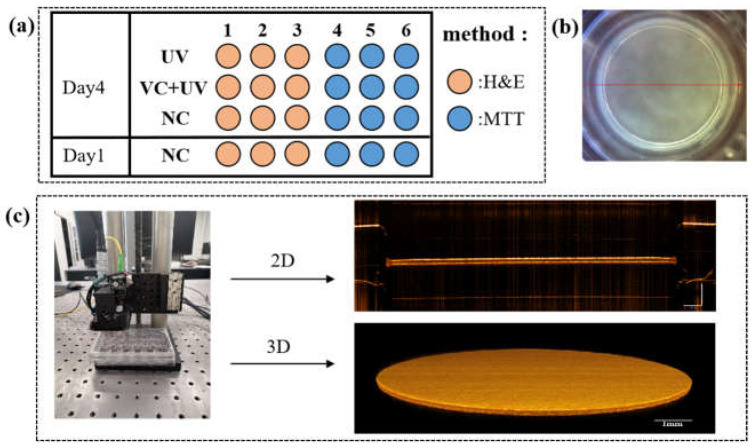
(**a**) Experimental groupings, MTT experiments, and H&E staining microscopic observation. UV—UVA irradiation group (Group 1); UV + VC—UVA irradiation group with medium VC (Group 2); NC—normal cultured AS (includes Group 3 and Group 4); (**b**) AS model images; (**c**) AS model for 2D and 3D OCT data acquisition.

In this study, we used the Skinovo epidermal AS model (Hangzhou Regenovo Biotechnology, Ltd., Hangzhou, China) and inoculated keratinocytes on transwells (Corning Inc., 3413, New York, NY, USA) for air–liquid culture. In the standard culture cycle for AS, the full differentiation of skin cells occurs on day 11. Thus, on day 11, the AS models were used in the photodamage experiments. A total of 24 air–liquid cultured AS models were adopted, and were equally divided into 4 groups, as shown in Figure 1a. Group 1 was the UV-irradiated group, where the AS models were transferred to a 24-well plate pre-spiked with phosphate-buffered saline (PBS) and were irradiated with UVA (365 nm) for 5 min daily (single irradiation dose of 3 J/cm^2^ which is a proper amount to induce skin damage in experiments). As shown in Figure 1b,c, each irradiation process was followed by a continued incubation cycle for 24 h before the OCT measurement was performed. A total of three irradiation and measurement cycles were performed. Group 2 was similar to the UV-irradiated group, but it was switched to an air–liquid culture containing 73 μg/mL of VC (an experimentally-determined dose that shows antioxidant effects while having no influence on cuticle differentiation) to verify the anti-photodamage efficacy of VC. This group had the same irradiation protocol as Group 1. Group 3 was a blank control (normal culture) that experienced the same OCT tests as Group 1 and Group 2, but without irradiation. After three irradiation cycles (day 4), as shown in Figure 1a, in order to verify the feasibility and accuracy of the OCT assay parameters for assessing AS photodamage, half of the models (*n* = 3 × 3) from each group were stained with H&E staining after the last OCT assay, and the model structure and cell number and status were observed using microscopy. The other half of the models (*n* = 3 × 3) were subjected to MTT experiments to measure light absorption values and analyze differences in tissue viability between groups. The other half of the models (*n* = 3 × 3) were subjected to an MTT assay to measure optical density (OD) and analyze the difference in tissue viability between groups. The six models in Group 4 were grown at the same time as the other models in the same medium. Half of the models were subjected to H&E staining (*n* = 3) and the other half to MTT experiments (*n* = 3) before the first irradiation, which were used to compare and analyse the status of the models before and after irradiation. We repeated the UVA irradiation experiment twice.

### 2.2. OCT Data Acquisition of AS

In this study, a self-developed spectral-domain OCT system was used. The system adopts a broadband light source with a central wavelength of 1310 nm and a full half-peak width of 248 nm. It has a Michelson interferometer type, and the signal is detected in parallel by a high-speed spectrometer. The system has an axial resolution of 3.5 μm and a lateral resolution of 13 μm in air, and is capable of imaging depths of up to 3.5 mm. The refractive index of AS tissue was set at 1.38 [35], corresponding to an axial resolution of 2.53 μm within the skin and an imaging depth of 2.59 mm. During the OCT imaging, AS models were sealed in sterile culture well plates, and the optical path difference and focal plane position of the OCT device relative to the AS remained consistent during all sample measurements. For each model at a time, 3D-OCT volume data of 10 mm × 10 mm × 2.59 mm (1000 pixels × 1000 pixels × 1024 pixels) were collected. AS models were detected using OCT before the first irradiation and 24 h after each UV irradiation. Thus, the OCT data at four different stages were obtained. The AS of Group 1 and Group 2 was irradiated with UVA irradiation at 24 h intervals, which was carried out three times in each group. The AS models were examined using OCT before the first irradiation and after each UV irradiation so that the OCT data were obtained for each group of AS models at four time points.

### 2.3. Multidimensional Parameter Quantification of AS Using OCT

#### 2.3.1. Quantitative Analysis of AS Thickness, Cuticle Thickness, and Surface Roughness

The thickness of AS is an important parameter for assessing the quality of AS. The condition of the AS cuticle is an important indicator of the degree of photodamage. The rate of cuticle differentiation, structural laxity, or firmness is reflected in the thickness of the cuticle, and the degree of cuticle surface fluctuation directly influences the surface roughness of AS. Therefore, cuticle thickness and surface roughness are important morphological parameters for detecting photodamage in AS. In order to quantify the changes in surface roughness and cuticle thickness of AS during UV irradiation, the skin surface profile first needs to be accurately extracted.

**Figure 2:**
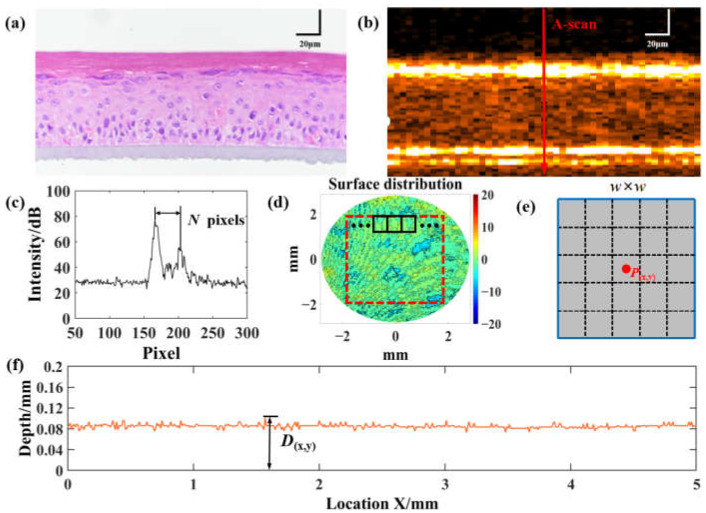
AS intensity signal analysis. (**a**) H&E staining for Group 4. (**b**) OCT intensity image. (**c**) OCT interferometric signal in (**b**). (**d**) AS 2D surface profile image. The area surrounded by the red border is the ROI. The area surrounded by the black border is the sliding average window. Sliding window method for calculating roughness of AS. (**e**) The size of the window is w pixel × w pixel. The center pixel point of the window is (x,y) and the average roughness inside the window is *s*_(x,y)_. The roughness of the central pixel point *P*_(x,y)_ is set to the average roughness within the window. (**f**) Image of 2D thickness distribution.

Figure 2a shows the H&E staining of AS after 11 days of air–liquid culture. It shows the structural information of AS. As can be seen, the artificial skin is fully differentiated on day 11. We can see the dark red cuticle in the upper layer of the AS and the keratinocytes inside the AS. The bottom gray layer is the basement membrane of the transwell. Figure 2b shows the corresponding OCT B-scan image. The two bright bands in the image are the cuticle and basement membrane of the AS. Figure 2c shows the A-scan intensity signal of the red arrow in Figure 2b. The two intensity peaks in Figure 2c correspond to the location of the bright band in Figure 2b, which represents the upper and lower surfaces of the AS. We can calculate the thickness from the number of pixels N between two intensity peaks to obtain the overall AS thickness distribution [31]. The surface roughness is determined by the upper surface profile of the skin. However, in the actual OCT measurement process, AS detachment from the basement film, uneven basement film, and tilted sample placement can lead to the overall tilting of the AS, and there is an overestimation bias in calculating the surface roughness using the upper surface profile. Therefore, we extracted the surface profile through the thickness value and flattened the lower surface indirectly to exclude interference. As shown in Figure 2d, we calculated the thickness of each A-scan and then subtracted the average thickness to obtain a 2D surface profile image. To eliminate the effect of noise, we used the sliding window method to calculate the skin surface roughness. The average skin roughness was calculated according to the ISO/FDIS 25178-2 standard definition [38,39], with the average roughness Sa defined as Equation (1):

(1) $$S_{a}=\frac{1}{PN}{\sum_{x=0,y=0}^{{(x-x_{0})}^{2}+{\left(y-y_{0}\right)}^{2}\leq r^{2}}s}_{\left(x,y\right)}$$

where x_0_ and y_0_ are the skin center locations, r is the radius of the target area and PN is the total number of sampling points in the target area. *s*_(x,y)_ is the corresponding average roughness at (x,y). It is calculated using the difference in height between each pixel point in the window and the average surface height of the skin, as shown in Figure 2e. It can be expressed by Equation (2):

(2) $$s_{\left(x,y\right)}=\frac{1}{w\times w}\sum_{x=0}^{d}\sum_{y=0}^{d}\left|D_{\left(x,y\right)}-\bar{D}\right|$$

(3) $$\bar{D}=\frac{1}{PN}\sum_{x=0,y=0}^{{\left(x-x_{0}\right)}^{2}+{\left(y-y_{0}\right)}^{2}\leq r^{2}}D_{\left(x,y\right)}$$

As shown in Figure 2f, *D*_(x,y)_ is the AS thickness at position (x,y). *D*_(x,y)_ = *N* ∗ Δ, where *N* is the number of pixels between the two intensity peaks of the A-scan [31]. Δ is the pixel size in the z direction. In this study, the average roughness of each sampling point was calculated using the sliding window method. The size of the sliding window is set to w pixel × w pixel, and $\bar{D}$ is the average surface height of the AS calculated by Equation (3).

#### 2.3.2. Quantitative Analysis of AS Texture Features

**Figure 3:**
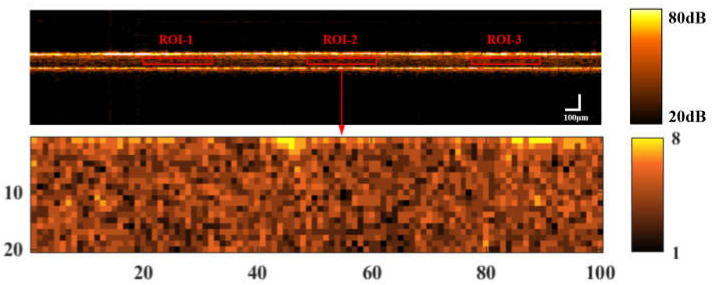
Excluding the cuticle and basement membrane, the 0.05 mm (z) × 1 mm (x) ROI (20 pixel (z) × 100 pixel (x)) was selected within the skin for texture characterization.

Texture is a visual feature that reflects homogeneous phenomena in an image, and OCT image texture comprises the arrangement of tissues in a skin section and the association between tissue areas. Texture information can be utilized to quantify changes in the micromorphological properties of AS tissue before and after UV irradiation. The gray-level co-occurrence matrix (GLCM) is widely used for image texture characterization [35,40]. In this study, GLCM analysis on 9 ROIs (regions of interest) from 3 B-scan images from both the central and peripherical areas within the skin was performed for image texture characterization, as shown in Figure 3.

First, the original OCT B-scan image was converted into a grayscale map with a gray level of *G* [40], which is

(4) $$g=\frac{g_{0}-g_{\min}}{g_{\max}-g_{\min}}\times G,$$

where *g* is the gray value of a pixel, *g*_0_ is the corresponding intensity value, *g*_min_ and *g*_max_ are the minimum and maximum intensity values in the whole original OCT image, and the gray level is set to *G* = 8. The GLCM is then generated from the grayscale image, and the probability of occurrence of the grayscale value pair (*i*, *j*), consisting of the pixel at (*x*, *y*) and the pixel with distance *d* at angle *θ* from (*x*, *y*), *P*(*i*, *j*, *d*, *θ*), is obtained as:

(5) $$P(i,j,d,\theta )=\left\{\left[\left(x,y),(x+\Delta x,y+\Delta y\right)\right]\right.\left|f(x,y)=i,f(x+\Delta x,y+\Delta y)\right.=\left.j\right\}$$

In the evaluation of *P* (*i*, *j*, *d*, *θ*), the ROI region was chosen as a 0.05 mm (z) × 1 mm (x) internal skin section (20 px × 100 px), the distance *d* = 3 pixels, and *θ* was set as 45 in order to reflect the image grayscale features for both the depth and horizontal directions.

Two common texture feature parameters, namely, the angular second moment (ASM) and correlation (COR) of skin images, were evaluated based on the GLCM results [35,40], which can be expressed as

(6) $$\left\{\begin{array}{l} \mathrm{ASM}=\sum_{i=1}^{8}\sum_{j=1}^{8}P^{2}(i,j,d,\theta )\\ \mathrm{COR}=\sum_{i=1}^{8}\sum_{j=1}^{8}i\cdot j\cdot P(i,j,d,\theta ) \end{array}\right..$$

Among the two parameters, the ASM reflects the degree of disorganization of the texture within the skin, and a small ASM indicates a disorderly image texture; on the other hand, COR reflects the consistency in the direction of the image texture regions. Analysis of internal skin features by GLCM extraction of image texture features was carried out to assess the effect of UV irradiation on the internal structure of AS.

#### 2.3.3. Characterization of AS Activity Using OCT Scattering Coefficients

**Figure 4:**
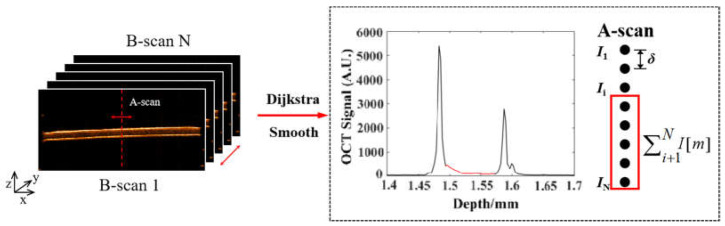
Modified depth-resolved attenuation coefficient quantification algorithm based on a single-scattering model. The red solid line indicates the area of intensity signal within the skin excluding the cuticle.

The optical properties of tissue play an important role in photodynamic therapy, tissue dynamics, and medical imaging monitoring [41]. From the perspective of tissue optics, AS tissue is composed of cells and extracellular matrices of different sizes and physiological states. Cells scatter incident light on the AS tissue. Thus, changes in tissue scattering coefficients indirectly characterize the state of cell survival, apoptosis, and necrosis within the tissue. Previous studies have also adopted sample scattering coefficients to characterize cell and tissue viability [41,42]. As shown in Figure 4, an improved depth-resolved (DR) algorithm was applied to the quantified AS scattering coefficient based on a single-scattering model. The scattering coefficient characterizes the tissue activity of AS and reflects the photodamage to the skin caused by long-term UVA irradiation.

The attenuation coefficient solved for in Beer’s law is equal to the sum of the scattering and absorption coefficients. In this study, the artificial skin tissue is a medium that highly scatters the 1310 nm NIR light, and its scattering is much greater than its absorption. Therefore, the attenuation coefficient calculated by Beer’s law can be approximated as the scattering coefficient. According to the Beer–Lambert law, the depth-resolved scattering coefficient for the non-uniform AS model can be expressed as follows [43]:

(7) $$\mu_{t}(z)=\frac{I(z)}{2\int_{z}^{\infty }I(z)dz}$$

where *μ*_t_ (*z*) represents the scattering coefficient at depth *z*, and *I* (*z*) represents the light intensity at depth *z* [43]. The DR algorithm is based on two assumptions. Firstly, the light is fully attenuated at the imaging depth. Secondly, the proportion of backscattered light in the attenuated light is a constant. Equation (7) is based on the assumption that the light is fully attenuated within the imaging depth. However, in real tissue imaging, there is background noise and incomplete attenuation of light. This would lead to the problem of underestimation in shallow places and overestimation in deep places if the scattering coefficient of AS is calculated directly based on Equation (7). In order to resolve the AS scattering coefficient quantization bias, an improved DR algorithm is proposed based on the analysis of the OCT interference signal of AS. We segmented the epidermal cell layer and removed the strong scattering signals from the AS cuticle and the basement membrane using Dijkstra’s algorithm [44]. As shown in Figure 4, the red signal segment in the signal plot on the right is the intensity signal corresponding to the active area within the skin (excluding the cuticle). This eliminates the bias for the underestimation of the AS scattering coefficients caused by strong background scattering noise. The fitted average scattering coefficient was used as the terminal scattering coefficient of the deep AS layer to recursively solve for the scattering coefficient of the epidermal cell layer. Thus, the overestimation bias of the AS deep scattering coefficient due to the incomplete attenuation of light within the AS model was eliminated.

As shown in Figure 4, there are two highlighted bands on the OCT B-Scan image of the AS. The uppermost highlighted band is the cuticle of the AS, which is composed of flat, dead cells without nuclei. The bottom highlighted band is the basement membrane of the air–liquid culture of the AS, and neither highlighted band is the living cell layer of the AS. The A-scan signal from OCT shows that the cuticle and basement membrane of the air–liquid culture of the AS show a clear bimodal distribution, while the OCT scattering signal after the removal of the bimodal peaks shows an exponential decay characteristic, as shown in the red signal area in Figure 4. Therefore, quantification of the scattering coefficient to characterize the active state of the AS tissue requires the removal of the upper surface (cuticle) and the lower surface (basement membrane). However, the overall thickness of the AS model is small, and the OCT signal is not fully attenuated within the AS model. If the DR algorithm in the paper [43] is used directly, it leads to an overestimation of the scattering coefficient in the deeper layers of the AS model. This bias is inherent to the conventional DR algorithm. This study was conducted to address this scattering coefficient overestimation problem. As shown in Figure 4, the exponential fit coefficient in the red signal region in Figure 4 was used as the scattering coefficient *μ*[*N*] for the deepest (last) pixel point of the AS model, and then the scattering coefficients for the other depth pixel points of the AS were recursively solved forward using *μ*[*N*] as the reference, which can be expressed as

(8) $$\mu [i]=\frac{I[i]}{2\delta \cdot \sum_{i+1}^{N}I[m]}\left(1-e^{-2\delta \cdot \sum_{i+1}^{N}\mu [m]}\right)$$

As shown in Figure 4, there was a small number of sampling points for the OCT signal within the AS model. To improve the accuracy of the exponential fit of the AS single-scattering model, we calculated the sliding average of the A-scan intensity signal in the x-z and y-z directions with a window size of 5 pixels. The average of the scattering coefficients of each pixel in the living cell layer inside the AS model was used to characterize the tissue viability of the AS.

### 2.4. H&E Staining

Each AS model was fixed in 4% formaldehyde for 2 h. After dehydration, paraffin embedding, and tissue sectioning, the samples were stained with hematoxylin–eosin (H&E), and histomorphological pictures of the AS were captured under a 40× microscope.

### 2.5. Tissue Viability Testing

The MTT experiment was used to test cell proliferation and cell activity. The principle of the assay is that special substances in the mitochondria of living cells reduce exogenous MTT (i.e., 3-(4,5-dimethylthiazol-2-yl)-2,5-diphenyltetrazolium bromide) to blue-purple crystals, which are insoluble in water and are deposited in the cells. Dead cells do not react with MTT. Therefore, the number of living cells correlates positively with the number of crystals deposited.

In the experiment, each AS model was placed in a 1 mg/mL MTT solution and incubated at 37 °C for 3 h. MTT was reduced by succinate dehydrogenase in the mitochondria of living AS cells to form water-insoluble blue-purple crystals (formazan) deposited in the cells. The isopropyl alcohol shaker was used by shaking at low speed to fully dissolve the blue-purple crystals (formazan). The absorbance value of each culture well was measured at OD570 nm using an enzyme marker (ThermoFisher, VARIOSKAN LUX, Singapore). The measured absorbance value OD indirectly reflects the number of viable cells within the AS model [45], which can be used as a characterization parameter for the tissue viability of the AS model.

Keratinocytes act as the main scattering particles within the artificial skin epidermis model. The density of scattering particles (keratinocytes) directly affects the scattering coefficient of the model. Comparing the MTT tissue viability test results with the OCT scattering coefficient quantification results, a link between the OCT scattering coefficient and AS activity can be established, and the scattering coefficient obtained from OCT non-destructive imaging can then be used to characterize the accumulated UVA photodamage.

### 2.6. Statistical Analysis

SPSS (v 25.0; SPSS Inc., Chicago, IL, USA) was used for statistical analysis [46]. Data were analyzed by calculating the mean and standard deviation of the parameters in each group of AS. One-way ANOVA (Welch’s test) was used to analyze the differences in morphological parameters between the three model groups, and a t-test was used to analyze the differences in scattering coefficients and optical density values (OD) between the different groups of AS models; *p* < 0.05 was considered a significant difference. Pearson correlation analysis was used to investigate the relationship between the scattering coefficient and skin activity, as well as the relationship between textural characteristic parameters and the UVA irradiation dose.

## 3. Results

### 3.1. Characterization of UVA Photodamage by AS Thickness, Cuticle Thickness, and Surface Roughness Using OCT

The cuticle is the end product of the cellular life cycle within the skin. When cells are shed, the dead cells in the basal layer are pushed to the skin surface to form the cuticle [27]. Continuous cumulative UVA irradiation promotes cell shedding from the skin, causing a loosening of the internal structure of the cuticle, the formation of a thicker cuticle, and surface skin roughness. In order to quantify the differences in thickness, cuticle thickness, and roughness between the pre-irradiated skin models and the groups after three irradiation cycles, statistical analysis was carried out using the mean of each group of models. Before irradiation (day 1), all AS models were cultured in the same way and had the same morphological structure. Therefore, all samples were averaged for statistical analysis. Before irradiation, the AS thickness distribution was between 88~92 μm, and the cuticle thickness distribution was between 19~25 μm. After three cycles of UVA irradiation (day 4), differences in morphological parameters between groups were observed, as listed in Table 1.

**Figure 5:**
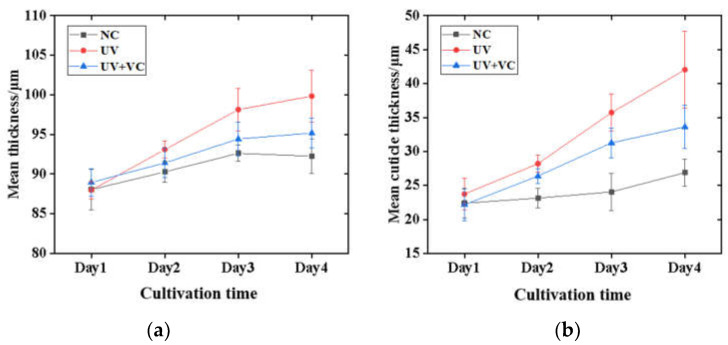
(**a**) Mean skin thickness for each group of AS with time; (**b**) mean cuticle thickness for each group of AS with time.

As shown in Figure 5a,b, three groups showed an increase in morphological parameters relative to the baseline (before irradiation) values and the increase in thickness of the AS was mainly due to the increase in the cuticle thickness. The photodamage was considered to be cumulative as the duration and dose of UVA exposure increased. The normal culture group (NC) had a skin thickness and cuticle thickness increase of 3~5 μm, which is in accordance with the normal growth state of the skin at the end of the air–liquid culture. The UV irradiation group (UV) showed a significant increase in skin thickness and cuticle thickness, around 15~20 μm, which can be explained by the effect of photodamage induced by UV irradiation. That is, through UVA irradiation, cell shedding was promoted and cells located in the basal layer were pushed upwards to form a thicker cuticle. To verify the feasibility of our method, a one-way analysis of variance (Welch’s test) was performed [46]. The results showed a significant difference in skin thickness between the three groups (*p* = 0.006 < 0.05) and a significant difference in cuticle thickness between the three groups (*p* = 0.000382 < 0.005).

**Figure 6:**
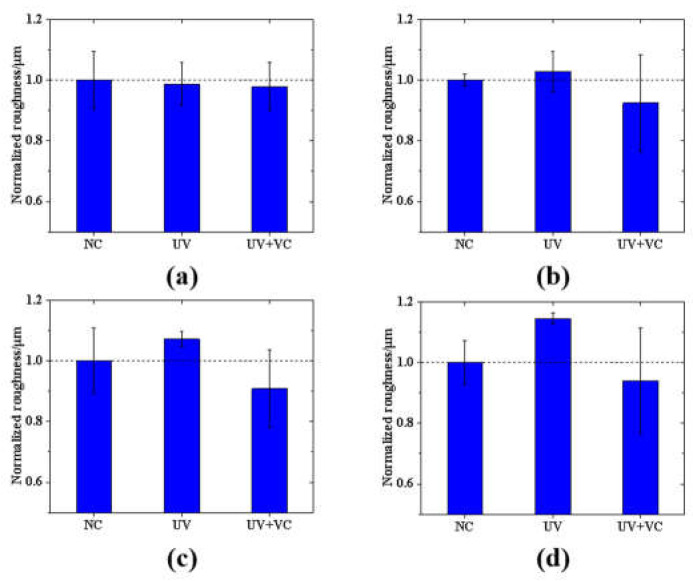
Variation in normalized values of model roughness with cycles of irradiation for three groups: (**a**) day 1 (before irradiation); (**b**) day 2 (after one irradiation cycle); (**c**) day 3 (after two irradiation cycles); (**d**) day 4 (after three irradiation cycles).

In this study, the AS surface contour point heights were obtained, and surface roughness was analyzed. The average surface roughness of the three groups of models was calculated at four different time points (day 1~4). The mean roughness of the NC group was used as the baseline value to normalize the remaining groups and to analyze the differences in model roughness between groups. As shown in Figure 6a, on day 1, the three groups of models had the same incubation process without UV irradiation, and the normalized roughness values were similar for all groups (NC: 1.00 ± 0.09 μm; UV: 0.98 ± 0.07 μm; and UV + VC: 0.97 ± 0.07 μm). As shown in Figure 6b–d, the difference between the normalized roughness values of the UV and NC groups increased with the number of irradiation cycles. The UV group had the largest Sa with a low variance (day 2: 1.02 ± 0.06 μm; day 3: 1.07 ± 0.02 μm; and day 4: 1.14 ± 0.02 μm), which clearly indicates the effect of UV irradiation on the AS surfaces. The UV + VC group had the lowest normalized roughness values of the three groups, but with a large distribution range (day 1: 0.92 ± 0.16 μm; day 2: 0.91 ± 0.13 μm; and day 3: 0.93 ± 0.17 μm).

We analyzed the difference in model roughness between the three groups at different incubation times (day 1–day 4) via an F-test. The results showed that there was no significant difference between the three groups of samples on day 1 (*p* = 0.299 > 0.05), whereas there was a significant difference between the three groups of samples on day 2 (*p* = 0.0011 < 0.05), there was a significant difference between the three groups of samples on day 3 (*p* = 0.0027 < 0.05), and there was a significant difference between the three groups of samples on day 4 (*p* = 0.0003 < 0.05). The results indicate that, on the first day of irradiation, although there was a difference in roughness between the three groups of samples, it was not significant. From day 2 to day 4, as the photodamage accumulated, the roughness of the three groups of samples showed significant differences.

### 3.2. Characterization of UVA Photodamage Based on Texture Features from OCT Detection of AS

**Figure 7:**
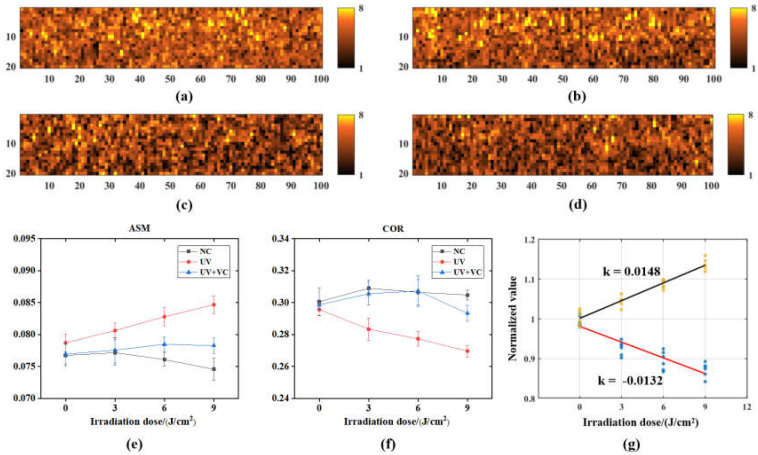
(**a**) Grayscale map of texture before irradiation; (**b**) grayscale map of texture of NC on day 4; (**c**) grayscale map of texture of UV on day 4; (**d**) grayscale map of texture of UV + VD on day 4; (**e**) variation in ASM texture parameters reflecting the internal structural looseness of the skin with incubation time; (**f**) variation in COR texture parameters reflecting the internal structural orientation consistency of the skin with incubation time; (**g**) linear fit of texture parameters of the UV group of AS with UVA irradiation dose.

In this study, we calculated the texture features of the skin based on the internal structure map of the AS detected by OCT. Long-term UVA irradiation leads to changes in the internal structure of the skin, which may be evaluated from the changes in the internal structure grayscale maps mentioned in Section 2.3.2. Typical grayscale maps for AS textures are shown in Figure 7a,b.

We calculated two texture features (ASM and COR) based on the GLCM method with multiple ROIs (0.05 mm (z) × 1 mm (x)) intercepted inside each AS model. ASM reflects the degree of disorganization of the image texture. COR reflects the directional similarity of the image texture, which can characterize the consistency of the direction of the layered structure within the AS. Figure 7e,f shows the variation in the AS texture parameters for the irradiation dose of each group. The ASM of the UV group continued to increase with time, as shown in Figure 7e, and, on day 4, it was 13.4% higher than that of the NC group (0.075 ± 0.002) and 9.0% higher than that of UV + VC group (0.078 ± 0.001). The COR of the UV group decreased with time, as shown in Figure 7f and was 12.9% lower than that of the NC group (0.31 ± 0.003) and 6.9% lower than that of the UV + VC group (0.29 ± 0.005) on day 4. The texture parameters of the UV group were normalized to the baseline values and linearly fitted to the UVA irradiation dose, as shown in Figure 7g. It can be seen that periodic cumulative UVA irradiation leads to a linear increase in the degree of internal skin texture disorganization (ASM) (Pearson’s correlation coefficient is 0.984) and a linear decrease in the consistency of skin texture orientation (COR) (Pearson’s correlation coefficient is −0.926).

### 3.3. H&E Staining to Verify the Accuracy of OCT Morphological Characterization

**Figure 8:**
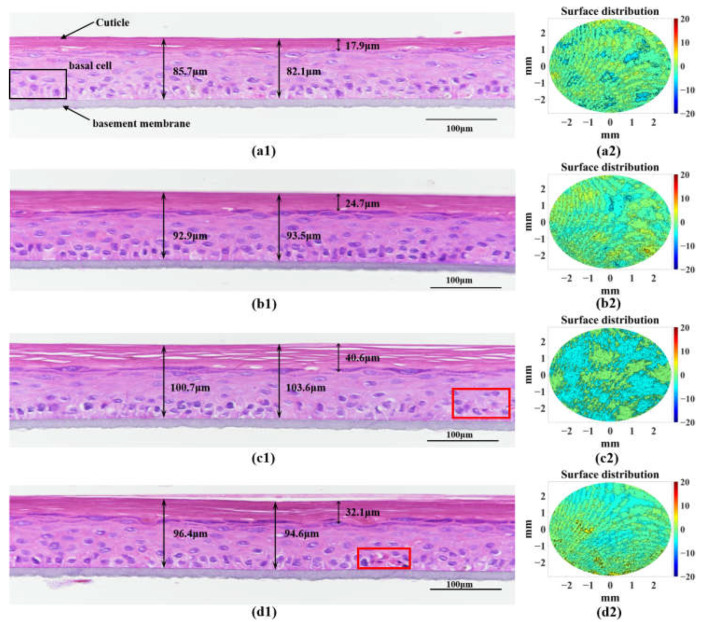
H&E staining microscopic pictures and surface profile maps. (**a1**–**d1**) H&E staining microscopic pictures of Before irradiation, NC-Day4, UV-Day4 and UV + VC-Day4; (**a2**–**d2**) Surface profile maps of Before irradiation, NC-Day4, UV-Day4 and UV + VC-Day4. The red sqare shows darkly stained clusters of apoptotic cells.

To verify the accuracy of OCT quantification of morphological parameters in characterizing UVA photodamage, we performed H&E staining microscopy experiments, as shown in Figure 8(a1–d1). The results of the H&E staining sections were consistent with the morphological results obtained from OCT measurements, with UV irradiation leading to an increase in AS thickness and cuticle thickness and a loosening of the cuticle structure. Figure 8(a2–d2) shows the surface profile maps corresponding to the AS model, with the UV group having a distinct surface profile. The surface profile maps obtained based on the AS thickness distribution reflect the roughness of the skin. The H&E staining results reveal that UVA irradiation promotes cell shedding and accelerates the rate of apoptosis in the skin. UVA irradiation caused the loose and disorganized structure of the internal cell layer of the skin and the loose structure of the surface cuticle, creating a thicker cuticle and surface skin roughness. This is highly consistent with the texture parameters within the epidermal layer (Figure 7), such as the thickness, cuticle thickness, and surface roughness (Table 1) obtained with the OCT measurements. The feasibility of OCT morphological parameter detection for the quantitative characterization of UVA-photodamaged skin structures was verified. The combined analysis of the difference in the degree of cuticle structure loosening in the AS model as measured by H&E staining and the results of surface roughness as quantified by OCT showed that UVA irradiation-induced cuticle structure loosening was the main cause of the increase in skin surface roughness. The addition of VC to the medium inhibits photodamage caused by UVA irradiation, maintains the structural stability of the cuticle, and reduces skin surface roughness.

### 3.4. Characterization of UVA Irradiation Photodamage by Tissue Optical Parameters of OCT

**Figure 9:**
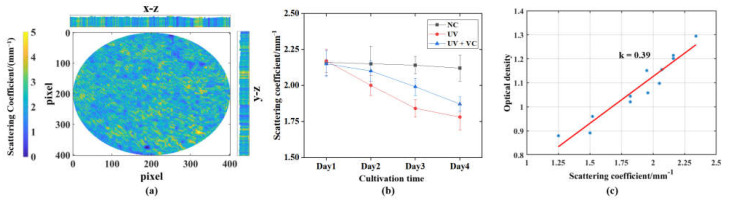
(**a**) The 2D distribution of scattering coefficients and B-scan scattering coefficient distribution in the x-z and y-z directions past the AS centroid; (**b**) OCT imaging characterizing the variation in scattering coefficients for each group of AS with incubation time and irradiation dose; (**c**) linear fit of scattering coefficients and optical density.

Morphological parameters and textural information reflect the effect of UVA irradiation on the internal and external structure of AS. However, the damage to the internal cells of the AS could not be characterized by this method. To characterize the damage of UVA irradiation on the cells inside the AS, we calculated the scattering coefficients for each AS model for four consecutive days based on the modified DR algorithm explained in Section 2.3.3. MTT assays were performed to characterize the tissue viability of the OD values before irradiation and after three irradiation cycles of AS, and the results are shown in Table 2. The correlation between the model scattering coefficients and the MTT assay results were analyzed. It can be seen that the scattering coefficients of the AS model are highly positively correlated with the tissue viability (i.e., OD) of the MTT assay (Pearson’s correlation coefficient is 0.986). As shown in Figure 9a, the internal pixel-level scattering coefficient of AS and the overall scattering coefficient distribution of AS can be obtained by improving the DR algorithm. The feasibility of quantifying the scattering coefficients of the AS to characterize the cell damage caused by UVA irradiation was also demonstrated in conjunction with the MTT experiment.

Figure 9b shows the variation in AS scattering coefficients for each group in relation to irradiation dose. We used the independent samples t-tests to analyze the differences in scattering coefficients and OD values between the models of the groups after irradiation and before irradiation [46]. Before irradiation, different groups showed a good constancy in their OD values with no significant difference in the t-test results (*p* = 0.326 > 0.05), and the scattering coefficient demonstrated the same behavior (*p* = 0.488 > 0.05). Since the AS models used had experienced full differentiation, their model state and cell numbers were stable. Thus, the scattering coefficient and OD of the NC group models also remained stable. The overall scattering coefficient of the NC group did not change significantly throughout the experimental period, with no significant difference in the scattering coefficient (day 4) compared to that before irradiation (day 1) (*p* = 0.375 > 0.05). The scattering coefficients of the UV and UV + VC groups continued to decrease with the accumulation of irradiation damage, eventually decreasing by 17.6% and 13.4%, respectively. The OD values of both AS models were significantly lower compared to pre-irradiation (*p* = 0.034 < 0.05, *p* = 0.006 < 0.05); moreover, the scattering coefficients were also significantly lower (*p* = 0.008 < 0.05; *p* = 0.007 < 0.05). Meanwhile, as the scattering coefficient was highly positively correlated with the OD value, we fitted the two linearly to obtain a mapping relationship between them, as shown in Figure 9c. We can quantitatively characterize photodamage using AS scattering coefficients in the bioactive dimension.

**Figure 10:**
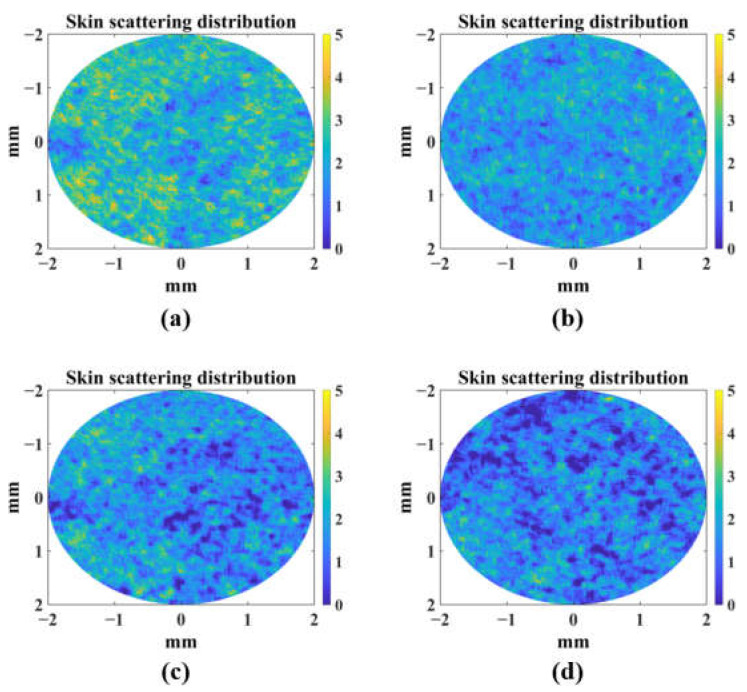
Scattering coefficient distribution of AS samples in UV group: (**a**) day 1; (**b**) day 2; (**c**) day 3; (**d**) day 4.

In this study, photodamage (UVA irradiation reduces the activity of AS, and, thus, the scattering coefficient shown in Figure 10 was detected using a scattering coefficient calculated from OCT intensity data using a modified DR algorithm.

## 4. Discussion

In this study, OCT was used to longitudinally monitor the structural parameters (i.e., morphological and textural characteristic parameters) and tissue optical parameters (i.e., scattering coefficient) of AS to evaluate the effect of photodamage on the skin. For the monitoring of AS morphological parameters, as shown in Figure 5, the growth rate of the cuticle thickness in the UV group was greater than that in the NC group due to photodamage. In addition, photodamage was also reflected in the AS surface roughness, as shown in Figure 6. As the photodamage accumulated, the roughness also continued to increase in the UV group compared to the NC group. These results are confirmed by the H&E staining results in Figure 8, where photodamage from UVA irradiation accelerated the shedding of cells within the skin to form a thicker cuticle, leading to a loosening of the skin’s keratinous structure and surface roughness. Therefore, it is possible to distinguish the degree of photodamage in different experimental groups of models based on the cuticle thickness and roughness. For the monitoring of AS texture characteristics, as shown in Figure 7, the ASM of the UV group increased linearly and the COR decreased linearly with the accumulation of photodamage. The results show that the extent of photodamage to the internal skin structure from long-term UVA irradiation can be assessed by the ASM to assess the disorganization of the internal texture and by the COR to assess the consistency of the internal texture orientation. As can be seen from Table 2, after three UVA irradiation cycles, the OD values of the UV group decreased significantly, indicating that continuous UVA irradiation caused a decrease in skin tissue viability and accelerated the rate of apoptosis within the skin, as shown in Figure 8c1. The scattering coefficient was highly positively correlated with the OD values during long-term irradiation, suggesting that we can characterize skin activity and, thus, assess photodamage through the scattering coefficient.

Comparing the results of the three model experiments, the anti-photodamage VC factor exerted a protective effect on the AS samples during long-term UVA irradiation. It inhibited the rate of apoptosis within the AS, delayed the proliferation of the cuticle and maintained the morphological and structural stability of the skin to some extent. The results of the cuticle assay in Figure 5 show that the cuticle in the UV + VC group was thinner (33.61 ± 3.21 μm) and closer to that in the NC group compared to that in the UV group (42.04 ± 5.67 μm), reflecting the role of the VC factor in inhibiting the abnormal thickening of the cuticle (31.5%) in the UVA irradiation environment, as it slowed down the rate of apoptosis due to photodamage. As illustrated in Figure 6, the addition of the VC factor slowed down the increase in roughness induced by photodamage, but there were still some AS samples with a roughness higher than the NC, which was caused by UV irradiation, indicating that the VC factor could not completely eliminate photodamage. The results of texture quantification of the three model groups showed that the addition of the VC factor inhibited the increase in the ASM and the decrease in the COR. This shows the repairing effect of the VC factor on the internal structure of photodamaged skin. The OD and scattering coefficient of the UV + VC group were higher than those of the UV group, indicating that the VC factor could protect the cells inside the skin and reduce the photodamage from UVA irradiation on the cells inside the model. In conclusion, the VC factor can alleviate the photodamage caused by UVA irradiation to the skin to some extent, but it cannot completely protect against this damage.

## 5. Conclusions

In this study, we performed UV photodamage experiments on artificial skin models and OCT technology was adopted to continuously detect the structural and tissue optical parameters of artificial skin models. A multiparametric quantitative characterization of photodamage was achieved. An in vitro test of artificial skin for UVA irradiation damage detection was successfully established, and the combined OCT assay, H&E staining, and MTT experiments demonstrated that the skin model with VC can resist UVA irradiation damage. The results show that UV photodamage causes structural damage to the skin surface, leading to an increase in cuticle thickness and roughness, as assessed with OCT data and verified by H&E staining microscopy. UVA irradiation also causes internal structural skin damage, and the texture data obtained from the OCT B-scan images showed a good correlation with the UV irradiation dose. This illustrates the potential of such parameters in reflecting internal photodamage from UVA irradiation. We further quantified photodamage at the cellular level, and the scattering coefficient of the AS model was obtained from the OCT intensity signal by a modified depth-resolved method. This correlates well with the optical density values from MTT experiments, revealing the utility of OCT scattering coefficients in the evaluation of cell viability in UVA photodamage. Hence, OCT is capable of being a non-destructive and continuous assessment method of photodamage on the surface and internal regions of the skin. In the future, we will study the effect of drug factors in UV photodamage repair using artificial skin models and the OCT monitoring method. This multidimensional photodamage detection method is also applicable in dermatological diagnoses and helps us to further understand the characteristics of the skin pathogenesis process.

## Figures and Tables

**Table 1:** Morphological changes in each group of AS before and after UV irradiation.

Morphological Parameter	Model Groups			
	Before Irradiation	NC-Day4	UV-Day4	UV + VC-Day4
Mean thickness (μm)	89.46 ± 2.46	92.24 ± 2.14	99.84 ± 3.29	95.17 ± 1.86
Mean cuticle thickness (μm)	22.37 ± 2.75	26.87 ± 2.00	42.04 ± 5.67	33.61 ± 3.21

**Table 2:** Scattering coefficient and MTT experimental results.

Parameter	Model Groups			
	Before Irradiation	NC-Day4	UV + VC-Day4	UV-Day4
Optical Density	1.20 ± 0.08	1.17 ± 0.02	1.05 ± 0.02	0.95 ± 0.02
Scattering Coefficient (mm^−1^)	2.16 ± 0.09	2.12 ± 0.16	1.87 ± 0.05	1.78 ± 0.09

## Data Availability

Not applicable.
